# Lung transplantation during the COVID-19 pandemic

**DOI:** 10.6061/clinics/2020/e1978

**Published:** 2020-05-18

**Authors:** Marcos N. Samano, Paulo M. Pêgo-Fernandes

**Affiliations:** Disciplina de Cirurgia Toracica, Instituto do Coracao (InCor), Faculdade de Medicina FMUSP, Universidade de Sao Paulo, Sao Paulo, SP, BR.

While Brazil is second only to the United States in terms of the absolute number of kidney and liver transplants, the number of lung transplantations remains modest. According to the Brazilian Registry of Transplantation, in 2019 only 106 lung transplants were performed across seven institutions. Three hospitals were responsible for 83% of these procedures, of which the Instituto do Coração (InCor) of HCFMUSP was responsible for 42% of these transplants. The reason for having only a few centers trained to perform lung transplantation lies in the complexity of the organ which is one of the most immunogenic organs. With only a few centers, patients with advanced lung disease have difficulty in seeking care. As of December 2019, 187 patients were on the waiting list for lung transplant, and over the course of the year, 39 patients died while still on the waiting list, which denotes a mortality rate of 21.2%. This demonstrates the severity of the situation; the demand for transplants is greater than the availability of organs. We wished to examine the expected effect of the new coronavirus disease 2019 (COVID-19) pandemic on lung transplantation?

COVID-19 is a disease caused by the severe acute respiratory syndrome coronavirus 2 virus (SARS-CoV-2) first reported in December 2019 in the city of Wuhan, China, and has spread rapidly around the world. Since the lung is the principal organ affected by COVID-19, the first concern for transplantation during this pandemic is to establish safe organ donation. How do we establish safety? Initial recommendations were based on the likelihood of donor infection and their exposure to known or probable cases within 14 days prior to donation. In that scenario, reverse transcription polymerase chain reaction (RT-PCR) tests for SARS-CoV-2 were desirable but not mandatory, along with chest computed tomography to assess pulmonary infiltrates suspicious of viral injury (1). However, as community transmission became more prevalent, ANVISA (Agîncia Nacional de Vigilância Sanitária - National Agency of Health Surveillance) established the need for RT-PCR tests for SARS-CoV-2 for all cadaveric donors. Based on discussions with experts around the world, we have decided to accept donors in the following situations: (1) SARS-CoV-2 negative RT-PCR within 24 hours before transplantation, (2) no history of COVID-19 or previous suspicion, (3) preferably with chest tomography showing no pulmonary infiltrates suggestive of acute injury in less than 24 hours before transplantation. With regards to the recipient, the nasal swab is collected however, as there is no time to wait for the results of the RT-PCR, we proceed with the transplant in recipients without symptoms, and with a negative chest tomography with no signs of recent pulmonary infiltrates.

The effect of the pandemic on the demand for multiple organ donors in the State of São Paulo was evident. By way of comparison, the transplant group at InCor receives an average of 60 notifications per month. In April 2020, there were 41 notifications, resulting in only two transplants per month: one patient prioritized in extracorporeal membrane oxygenation (ECMO) and another type AB recipient.

Ethical considerations of performing transplantation during this pandemic must also be examined, because despite all the recommended care previously mentioned, the risk of COVID-19 in transplant patients remains uncertain. In a report by Pereira et al., of the 90 patients with solid organ transplantation in New York City who tested positive for SARS-CoV2, 68 required hospitalization and 16 patients (18%) died of the disease. Among the 90 transplant patients, 17 received lung transplants of whom 41% had severe manifestations of the disease. There is no information on the mortality in this subgroup (2). In contrast, Aigner et al., based on his own experience of a single transplant recovered after infection suggests the satisfactory evolution of immunosuppressed patients infected with COVID-19 (3).

Without being able to predict the effect of COVID-19 in immunosuppressed patients, defining the ideal recipient in this situation is complex. The capabilities of the hospital and its intensive care beds must also be considered. Assuming there is transmission control and adequate human and material resources, the transplant community believes that the program should be conducted normally. However, patients who are stable, with no progression of the disease and, and can wait for three to six months, must be educated on the risks of transplantation during this pandemic. In a webinar promoted by the International Society for Heart and Lung Transplantation (ISHLT) with specialists from different countries, the need to perform transplantation in patients at a greater risk of respiratory decline, such as patients with rapidly evolving pulmonary fibrosis or pulmonary hypertension, was clear. If the in-hospital scenario worsens, for example, increase in-hospital transmission and depletion of resources, transplants can be restricted only to those patients in emergent need who have a risk of imminent death (priority) and who are on mechanical ventilation or under care (ECMO).

The safety of the transplant team and the professionals involved must also be a priority at this stage. Despite the results of the RT-PCR for SARS-CoV-2 in both donors and recipients, the use of suitable personal protective equipment (PPE) in the organ procurement and implantation process is essential, since airway manipulation during lung transplantation is continuous.

What has been observed in Europe is a global reduction in the number of lung transplants, both due to the decrease in the number of donors, and the exhaustion of the health system. There is still no information on the impact of COVID-19 on lung transplantation in the United States. However, there are several transplant Centers in US and once the spread of the pandemic has not been uniform, some centers are still maintaining their transplant programs close to normal. The outlook that is expected in Brazil should be similar to what has been occurring in Europe, but further impacted by the fact that few centers account for almost all lung transplant procedures. Any effect on one of these centers will have a very large impact on this modality and from what has been observed so far, there will certainly be fewer donors, and fewer transplants in the coming months.

As this crisis is undoubtedly going to expose the level of preparedness of the health care system in Brazil, the need to encourage more lung transplant centers is evident, so that patients in need are not obliged to travel away from their home cities in search of such an exclusive modality. Given that the lung transplantation programs are poorly funded, both by the Unified Health System (SUS) and by insurance companies, only seven teams are registered in Brazil to perform this procedure. It is worth mentioning that although Brazil has the largest public transplant program in the world which started in 1989, the National Supplementary Health Agency (ANSS) does not yet recognize this modality within the list of procedures to be covered by health operators. For comparison, this activity is well funded by the Insurance Companies in US where there are 69 active programs registered with UNOS (2,652 transplants performed in 2018) and 174 programs registered on the ISHLT platform, responsible for 4452 lung transplants performed around the world annually.

Thus, at a time when COVID-19 cases are increasing on a logarithmic scale with more than 90,000 cases and 6,329 deaths in Brazil (30,374 cases and 2,511 deaths in the State of São Paulo)^1^, it is expected that the number of lung transplantations will significantly increase.

## Footnotes

1 As of May 1^st^, 2020. Available at http://www.covid.saude.gov.br
